# Genetic Diversity and Association Characters of Bacteria Isolated from Arbuscular Mycorrhizal Fungal Spore Walls

**DOI:** 10.1371/journal.pone.0160356

**Published:** 2016-08-01

**Authors:** Gopal Selvakumar, Ramasamy Krishnamoorthy, Kiyoon Kim, Tong-Min Sa

**Affiliations:** Department of Environmental and Biological Chemistry, Chungbuk National University, Cheongju, Chungbuk, Republic of Korea; USDA Forest Service, UNITED STATES

## Abstract

Association between arbuscular mycorrhizal fungi (AMF) and bacteria has long been studied. However, the factors influencing their association in the natural environment is still unknown. This study aimed to isolate bacteria associated with spore walls of AMF and identify their potential characters for association. Spores collected from coastal reclamation land were differentiated based on their morphology and identified by 18S rDNA sequencing as *Funneliformis caledonium*, *Racocetra alborosea* and *Funneliformis mosseae*. Bacteria associated with AMF spore walls were isolated after treating them with disinfection solution at different time intervals. After 0, 10 and 20 min of spore disinfection, 86, 24 and 10 spore associated bacteria (SAB) were isolated, respectively. BOX-PCR fingerprinting analysis showed that diverse bacterial communities were associated to AMF spores. Bacteria belonging to the same genera could associate with different AMF spores. Gram positive bacteria were more closely associated with AMF spores. Isolated SAB were characterized and tested for spore association characters such as chitinase, protease, cellulase enzymes and exopolysaccharide production (EPS). Among the 120 SAB, 113 SAB were able to show one or more characters for association and seven SAB did not show any association characters. The 16S rDNA sequence of SAB revealed that bacteria belonging to the phyla Firmicutes, Proteobacteria, Actinobacteria and Bactereiodes were associated with AMF spore walls.

## Introduction

The intimate symbiosis between plants and microorganisms trigger many functions which are ecologically important. Arbuscular mycorrhizal fungi (AMF) are obligate biotroph that form mutualistic symbiosis with 80% of land plants [1]. AMF are known to improve plant growth by enhancing nutrient uptake and in turn receive carbon from the host plants [2]. Many soil microorganisms living in mycorrhizosphere (rhizosphere and mycosphere) interact with AMF either synergistically or adversely. Initially, Bonfante-Fasolo and Vian [3] showed microscopic evidence of bacterial mediated AMF spore wall outer layer lysis. Later, Levy et al. [4] reported that the bacteria can even invade the spore wall layers and survive in the spore cytoplasm of AMF as endosymbionts. Several studies have reported that diverse bacterial communities were associated with AMF spores [5–7]. Bacteria belong to genera *Burkholderia*, *Pseudomonas*, *Variovorax* and *Chromobacterium* were more frequently associated with AMF spores of *Rhizophagus intraradices* (formerly—*Glomus intraradices*) [5] and fungal mycelium in the rhizosphere and non-rhizosphere soil. Scheublin et al. [8] reported that Oxalobacteraceae family was more specifically associated with AMF hyphal surface. Although these studies help to understand the bacterial communities associated with AMF spore, none of these studies explained how these bacteria were able to associate with AMF spores.

Arbuscular mycorrhizal fungi spores consist of two or more spore walls and the number of spore wall layers vary among different genera [9]. Bonfante-Fasolo and Grippiolo [10] identified the principal components of the AMF spore outer layer and reported that the spore wall contains chitin, glucosamine, proteins and polysaccharides. Biopolymer-degrading bacteria present on the spore outer hyaline layer could possibly utilize oligomers of chitin as nutrient source for their survival [11]. The spore associated bacteria have been isolated from different portions of AMF including spore cytoplasm [12], surface sterilized spores [6,13,14] and spore hyphae [8,15,16] and were reported to stimulate fungal growth. The interaction between AMF and bacteria have been shown to increase plant growth by enhancing phosphate solubilization [17], fixing atmospheric nitrogen [18], improving AMF spore germination and plant root colonization [12].

Thus, understanding the association between AMF and bacteria might contribute significantly in selecting suitable spore associated bacteria for improving AMF performance and bacteria survivability in introduced environment. The present study attempts to understand the association characters of AMF spore wall associated bacteria. Thus, we framed our objective to cultivate the bacterial communities associated with *Funneliformis caledonium*, *Racocetra alborosea* and *Funneliformis mosseae* and identify their characters for spore wall association.

## Materials and Methods

### Sample collection and spore isolation

The experiments in this study did not involve endangered or protected species. Soil samples were collected from salt affected reclamation land of Saemangeum in South Korea (35° 46’ 14.3” N and 126° 37’ 11.0” E). No specific nor special permission from the government was required for this location. The government of South Korea through the National Research Foundation projects allowed researchers access to the area since it aimed to improve the utilization of the area for agricultural purposes. Saemangeum is one of the world largest reclamation sites where plant growth and establishment were inhibited due to unequal distribution of soil salinity and low nutrient content [19]. Since it is a newly reclaimed area, there were no agricultural practices nor any other human activity that may have disturbed the nature and microorganisms present in the soil. The reclaimed land was dominated by natural grass plants such as *Phragmites australis*, *Cyperus polystachyos* and *Miscanthus sinensis*. *Phragmites australis* known as common reed is a large perennial grass and commonly found in the wet lands. *Cyperus polystachyos* is a weed plant and widespread in tropical and subtropical areas around the world, sometimes extending its range to temperate regions. *Miscanthus sinensis* belong to grass family and native to eastern Asia including South Korea and they can even survive under high stress environments.

Each rhizosphere soil sample (10 cm radius and 15 cm depth; approximately, one kg for each sample) was collected from dominant plant species along with their roots in a sterilized polybag and kept in icebox and immediately transported to the laboratory. Soil chemical properties such as pH, organic matter content (OM), available phosphorus (Av.P_2_O_5_) and total nitrogen were measured using standard laboratory protocols. The EC values of the soil samples varied from 0.13 to 36.5 dS/m, and an average pH of 6.7 (S1 Table). The soil contained an average of 4.1 g/kg OM, 0.026% total nitrogen, 32.6 mg/kg phosphorous and 0.56 cmol^+^/kg sodium. Spores were isolated by wet sieving and decanting method as described in Daniels and Skipper [20] followed by sucrose centrifugation as described in Utobo et al. [21].

### Spore morphological differentiation and molecular identification

Isolated spores were differentiated based on their morphological characters such as size, color, sporogenous cell and number of spore wall layers. They were grouped into three types namely Type 1- small (>106 μm), globose, dull yellow; Type 2 –big (>250 μm), globose, white and Type 3 –small (>106 μm), irregular, red brown (S1 Fig) based on the earlier descriptions of Bharadwaj et al. [22]. For molecular level identification of the grouped spores, five healthy spores from each type were taken in a microcentrifuge tube and surface sterilized with 2% chloramine-T and 100 μg/ml streptomycin (modified from Levy et al. [4]) for 30 min. The surface sterilized spores were transferred to a sterilized PCR tube containing 10 μl of 1:1 ratio of 10X PCR buffer and sterilized distilled water [23]. Spores were aseptically crushed with a sterilized blunt end Pasteur pipette to collect spore DNA. The 18S rDNA of arbuscular mycorrhizal fungal spores were amplified using nested PCR [24]. In the first PCR, GeoA2 and Geo11 primers were used to amplify the universal eukaryotic genes. In the second round of PCR, AMF specific primers AM1 and NS31 were used. The final product of the second PCR was directly sequenced and the closest neighbor was identified by searching against the genes in NCBI. The nucleotide sequence of 18S rDNA were deposited in GenBank under the accession numbers of KJ792101, KJ792102 and KJ792103.

### Spore associated bacteria (SAB) isolation

The morphologically differentiated spores were immediately used for SAB isolation to minimize the loss of bacteria during spore storage. Approximately 240 spores for each differentiated spore type were collected and vortexed for 5 s to remove soil debris. Then the spores were transferred aseptically to four spin columns modified with nylon net filter (30 μm) placed in 1.5 ml collection tube. Each column contained approximately 60 spores for each differentiated spore type. Disinfection solution was added to the spin column until the spores were fully submerged into the solution. After 0, 10, 20 and 30 min of disinfection treatment, the spores were washed with sterilized distilled water for 5–7 times. Sterile distilled water was used for 0 min disinfection. The final washings of each treatment were collected in a separate sterilized microcentrifuge tube. The efficiency of the spore surface sterilization was confirmed by plating the final washings in 0.08% nutrient agar (Merck KGaA) and PYEA (5% polypeptone and 1% yeast extract) media. To further confirm the efficiency of spore surface sterilization and the presence of bacteria on spore wall, the disinfected spores were viewed under scanning electron microscope (SEM). Briefly, the surface sterilized spores at different time intervals were aseptically transferred to cover slips, fixed using 20 μl sterile distilled water and air dried. After coating with gold, the spores were examined under SEM (Hitachi S-2500C, Hitachi Co., Japan).

Spores, surface sterilized with disinfection solution at different time intervals, were transferred aseptically to petri plates containing nutrient agar media and PYEA media in three replicates and incubated at 28±2°C for two weeks [12]. The bacterial colonies growing around the spores were purified and subcultured.

### Genetic diversity of SAB by BOX-PCR

Genomic DNA of SAB isolated from each spore type at different disinfection time interval were amplified directly using colony PCR or by isolating genomic DNA using genomic purification kit (Promega, USA). The primer used for the BOX-PCR reaction was BOX-AIR (5’-CTACGGCAAGGCGACGCTGACG-3’) as described in Naik et al. [25] with modifications. Each reaction volume of 20 μl contained 2 μl dNTP (10 mM), 2 μl of 10X PCR buffer, 2 μl primer (10 pmol), 0.2 μl Taq (1 unit) and 13.8 μl sterilized distilled water. The PCR program consisted of the following steps: initial denaturation at 94°C for 5 min for colony PCR and 3 min for genomic DNA template, next step consisted of 30 cycles of denaturation at 94°C for 3 s and 92°C for 30 s, annealing at 50°C for 1 min and extension at 65°C for 8 min and the final extension was at 65°C for 8 min. Cluster analysis were performed using dice coefficient model with unweighted pair grouping with mathematic average (UPGMA) in BioNumerics 7.1 software (Applied Maths, Belgium).

### Gram staining and spore associated characters of SAB

All SAB isolated from each spore type at different disinfection time interval were tested for Gram staining and spore association characters. Gram staining was performed using Gram staining kit and the bacterial suspensions were examined under upright microscope (Olympus CX41) for cell morphology. Qualitative chitinolytic activity was determined on plates containing colloidal chitin as described in Liu et al. [26]. Quantitative estimation of chitinase production was assayed as described in Rojas-Avelizapa et al. [27]. Cellulase production of SAB were evaluated on CMC agar plates as described in Kasana et al. [28]. Protease activity was determined by growing the bacteria in skim milk agar plates. Exopolysaccharide (EPS) production of SAB was determined by phenol-sulfuric acid method as described in Albalasmeh et al. [29].

### 16S rDNA sequencing and plant growth promoting (PGP) characters of SAB

16S rDNA of SAB was amplified using the universal bacterial primers 27F and 1492R [30]. Nearly complete 16S rDNA were aligned and the closest identities were obtained by searching against the type strain genes in EzTaxon. Phylogenetic dendrogram of SAB and its closest neighbors was constructed using neighbor-joining method after multiple alignment with CLUSTAL-W. The evolutionary distance between the isolates were calculated according to Jukes and Cantor model [31] and bootstrapped with 1000 replications using MEGA 6 software [32]. The nucleotide sequences were submitted to the NCBI GenBank with the accession numbers KM507113 to KM507163. The PGP characters of SAB was analyzed using the previously established protocols [33].

### Statistical analysis

Percentage data were subjected to arcsine square root transformation before analysis of variance (ANOVA) was performed. The mean significant difference was compared by Duncan’s Multiple Range Test (DMRT) at *P*≤0.05 using SAS package, Version 9.4.

## Results

### Spore identification and SAB isolation

**Fig 1:**
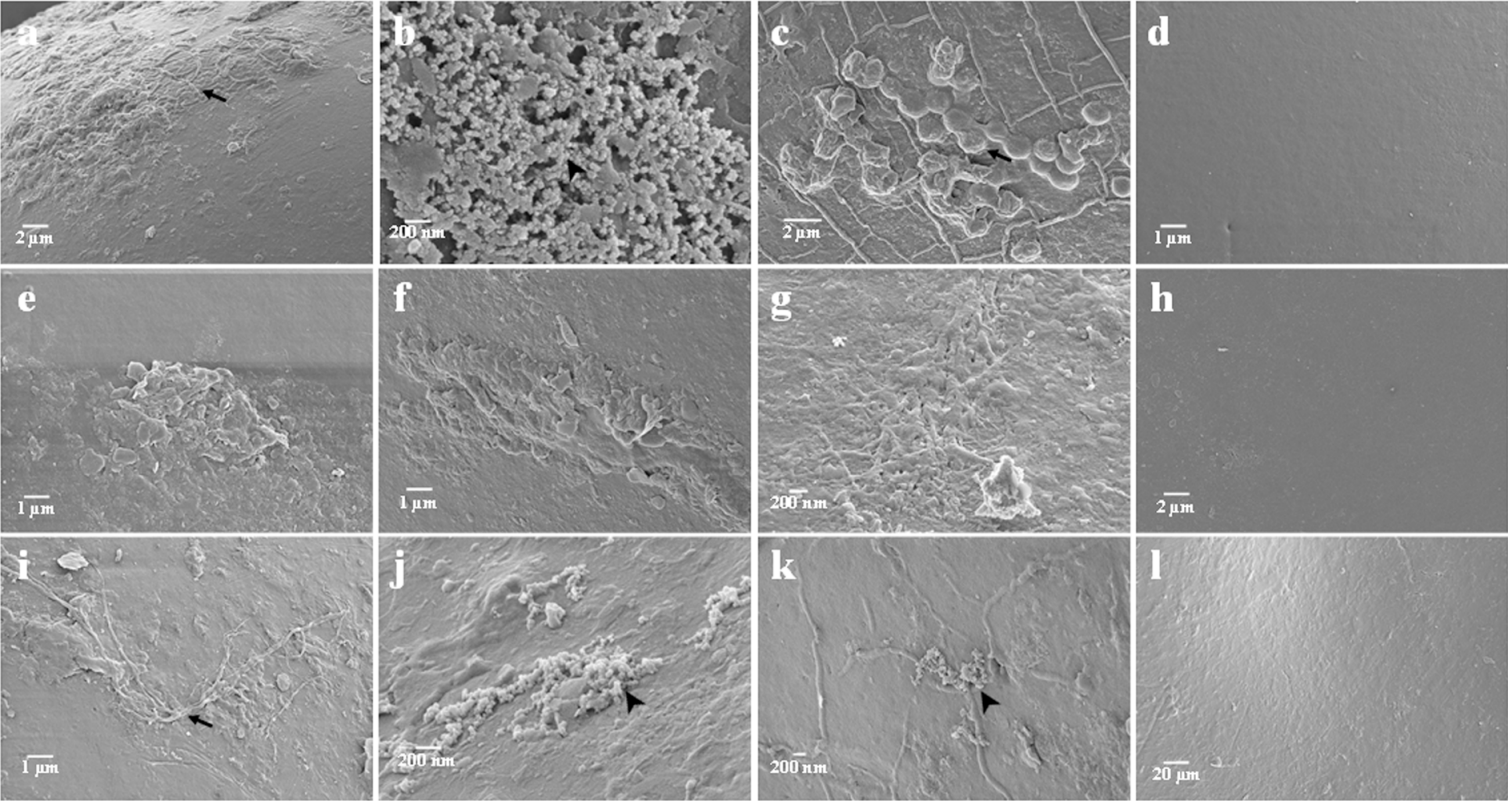
Spore after surface sterilization with disinfection solution at different duration. a, b, c, d—Type 1 spore after surface sterilization at 0, 10, 20 and 30 min. e, f, g, h—Type 2 spore after surface sterilization at 0, 10, 20 and 30 min. i, j, k, l—Type 3 spore after surface sterilization at 0, 10, 20 and 30 min. Arrow marks indicate bacterial cells of various shapes adhering to the spore wall; filamentous bacterial cells (a, i), coccus-shaped bacterial cells (c). Arrowhead indicates the formation of mucilaginous product replacing outer hyaline layer (b, j, k).

Morphologically differentiated spores were identified at the species level by molecular identification. 18S rDNA sequencing of spores revealed that Type 1 spore was closely identical (99%) to *F*. *caledonium*, Type 2 spore was identical (100%) to *R*. *alborosea* and Type 3 spore was identical (100%) to *F*. *mosseae* (S2 Table). To confirm the efficiency of spore surface sterilization, spores were viewed under SEM (Fig 1, S2 Fig). A total of 120 SAB were isolated from three different types of spore at different disinfection time interval. From *F*. *caledonium*, 16, 5 and 3 bacteria were isolated after 0, 10 and 20 min disinfection, respectively (S3 Table). From *R*. *alborosea*, 46, 15 and 5 bacteria were isolated after 0, 10 and 20 min disinfection, respectively. From *F*. *mosseae*, 24, 4 and 2 bacteria were isolated after 0, 10 and 20 min disinfection, respectively. There was no bacterial growth in completely surface sterilized spores at 30 min disinfection.

### Genetic diversity of SAB

**Fig 2:**
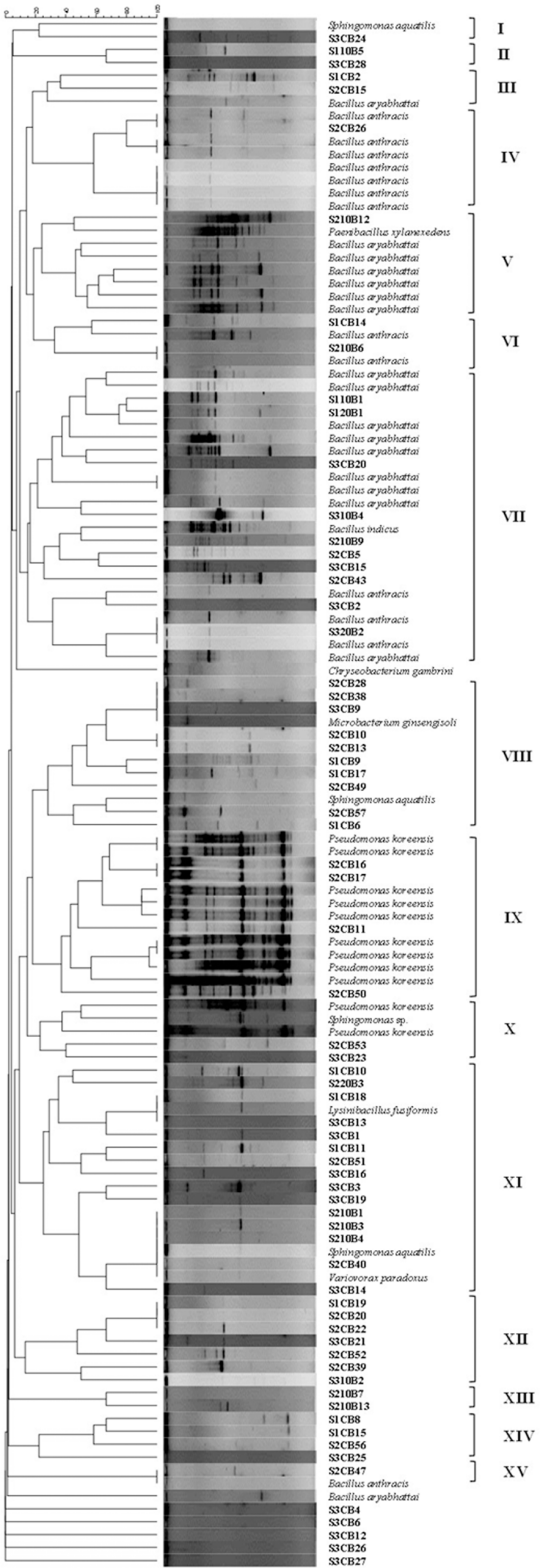
Dendrogram showing the genetic relationship as estimated by cluster analysis of BOX-PCR products of spore associated bacteria isolated form the spore walls of AMF. SAB isolated from all three types of spores at different time interval disinfection were analyzed. Dendrogram was constructed using the Dice coefficient with UPGMA algorithm.

To understand the genetic relationship between the cultivated bacteria from all three different spores, BOX-PCR fingerprinting method was approached. The molecular typing of BOX-PCR generated well resolved banding pattern with 1–11 amplification products and the band size varied from 100 bp to over 3000 bp (Fig 2). For the total of 120 SAB, 15 major clusters were formed and the genetic similarity between the clusters shared less than 20%. Among the 15 clusters, cluster II shared the maximum of 65% similarity with other clusters followed by cluster IV which shared a similarity of 40%. The remaining clusters shared about 20 to 30% similarities.

Bacteria associated with each spore type were analyzed separately to understand the genetic similarity of SAB within single AMF species. In *F*. *caledonium*, the banding pattern of SAB were grouped into 7 major clusters and each clusters shared only 30% similarity with the other clusters (S3 Fig). In *R*. *alborosea*, 9 major clusters were formed and the genetic similarity between the clusters were 20% (S4 Fig). In *F*. *mosseae*, the banding pattern of SAB formed 6 major clusters and cluster III had the maximum similarity of 40% with the other clusters (S5 Fig). The low similarity between the clusters in each spore type suggests that genetically diverse bacterial communities were associated with each AMF species.

### Gram staining and spores associated characters of SAB

**Fig 3:**
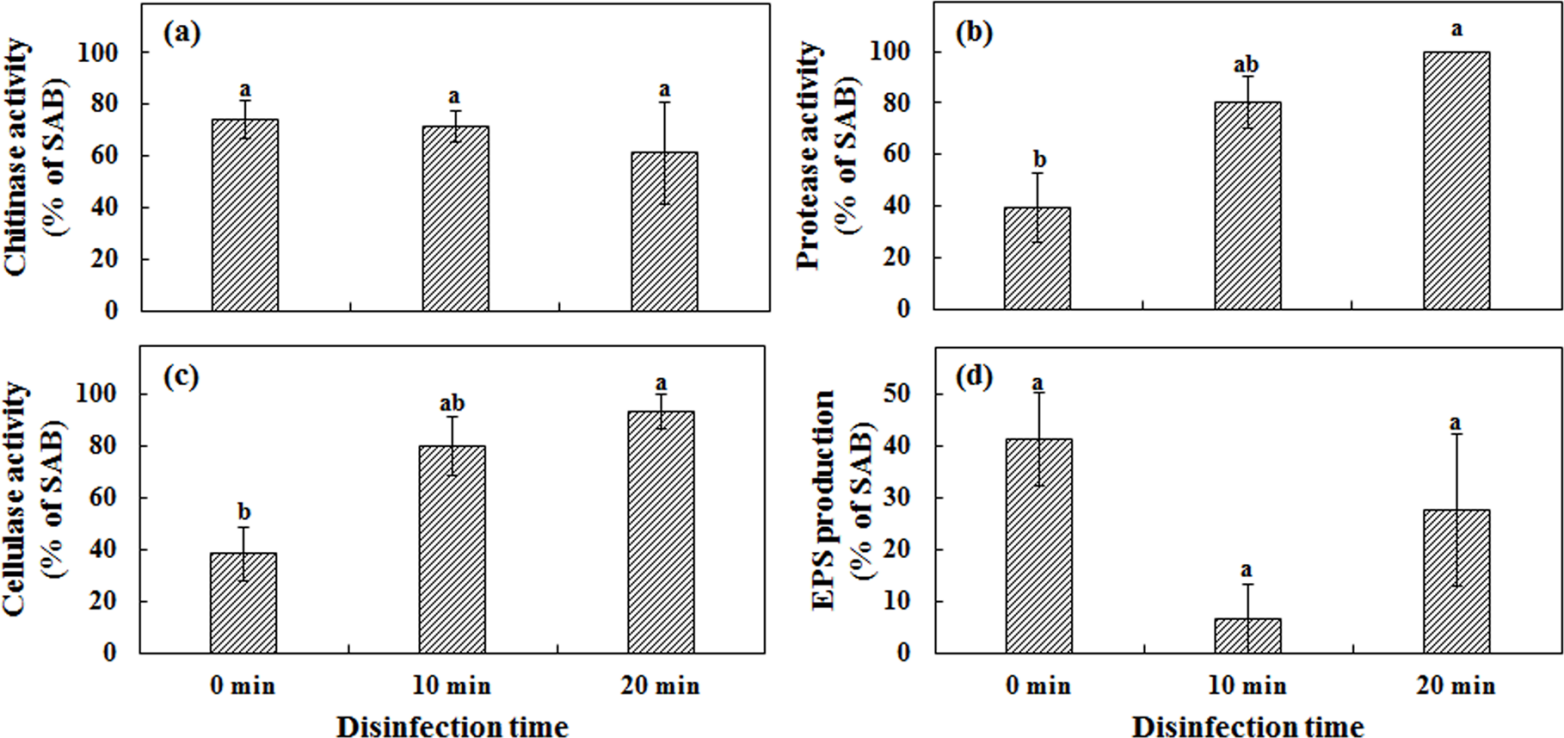
Spore associated characters of SAB. (a)–chitinase activity; (b)–protease activity; (c) cellulase activity; (d) EPS production. Means with same letter do not differ significantly according to DMRT test at *P* < 0.05 (arcsine transformed data).

The percentage of Gram positive bacteria isolated after 10 min and 20 min disinfected spores were significantly higher compared to SAB isolated from 0 min disinfected spores (S6 Fig). Chitinase activity of SAB isolated from 10 and 20 min disinfected spores did not differ from SAB isolated from 0 min disinfected spores (Fig 3A). However, irrespective of the disinfection time, majority of the SAB (60%) isolated from different disinfection time interval showed chitinase activity suggesting that chitinolytic enzyme activity is important for bacteria to strictly associate with AMF spores. Among the total of 86 SAB isolated from 0 min disinfected spores, only 39% of SAB showed protease activity (Fig 3B). The percentage of SAB which showed protease activity were significantly higher in 20 min disinfected spores suggesting that protease activity might help bacteria to strictly associate with AMF spores.

Cellulase activity followed the same trend with protease activity where significantly higher percentage of SAB showed cellulase activity isolated from 20 min disinfected spores (Fig 3C). The percentage of SAB which showed cellulase activity were higher at 10 and 20 min disinfected spores suggesting that cellulase enzyme activity play a major role in AMF and bacteria association. The high percentage of SAB that showed EPS production at 0 min disinfected spores than SAB isolated from 10 or 20 min disinfected spores suggests that EPS production might help in the initial association between AMF spores and bacteria (Fig 3D).

**Fig 4:**
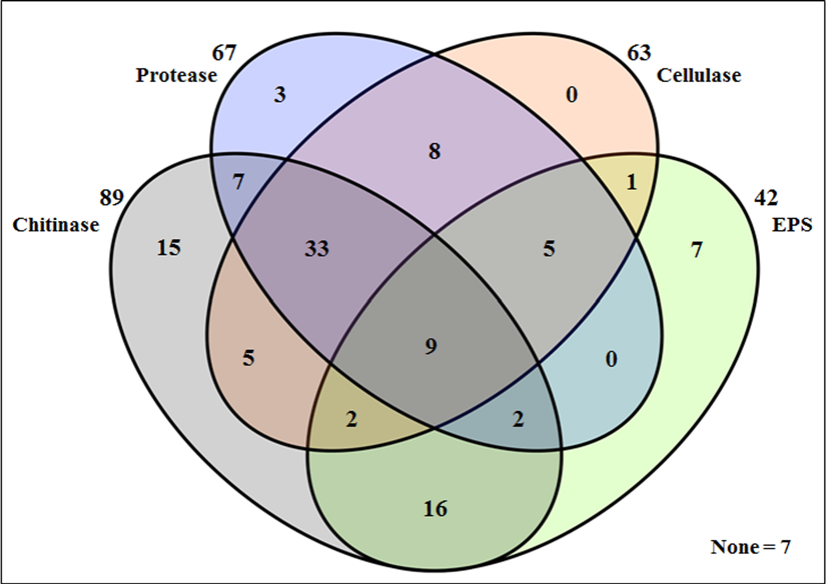
Venn diagram showing the spore association characters of SAB isolated from 0, 10 and 20 min disinfected spores.

To further understand the spore association characters of SAB, bacteria isolated from different disinfection time interval were grouped based on their association characters. Among the total of 120 SAB, 113 SAB were able to show one or more characters for association (Fig 4). The remaining seven bacteria which did not show any of the association characters were isolated from 0 min disinfected spores. SAB isolated from 10 min disinfected spores showed a minimum of one or more characters for association whereas SAB isolated from 20 min disinfected spores showed a minimum of two or more characters for association suggesting that bacteria having multiple association characters can have close association with AMF spores.

### 16S rDNA sequencing of SAB

**Fig 5:**
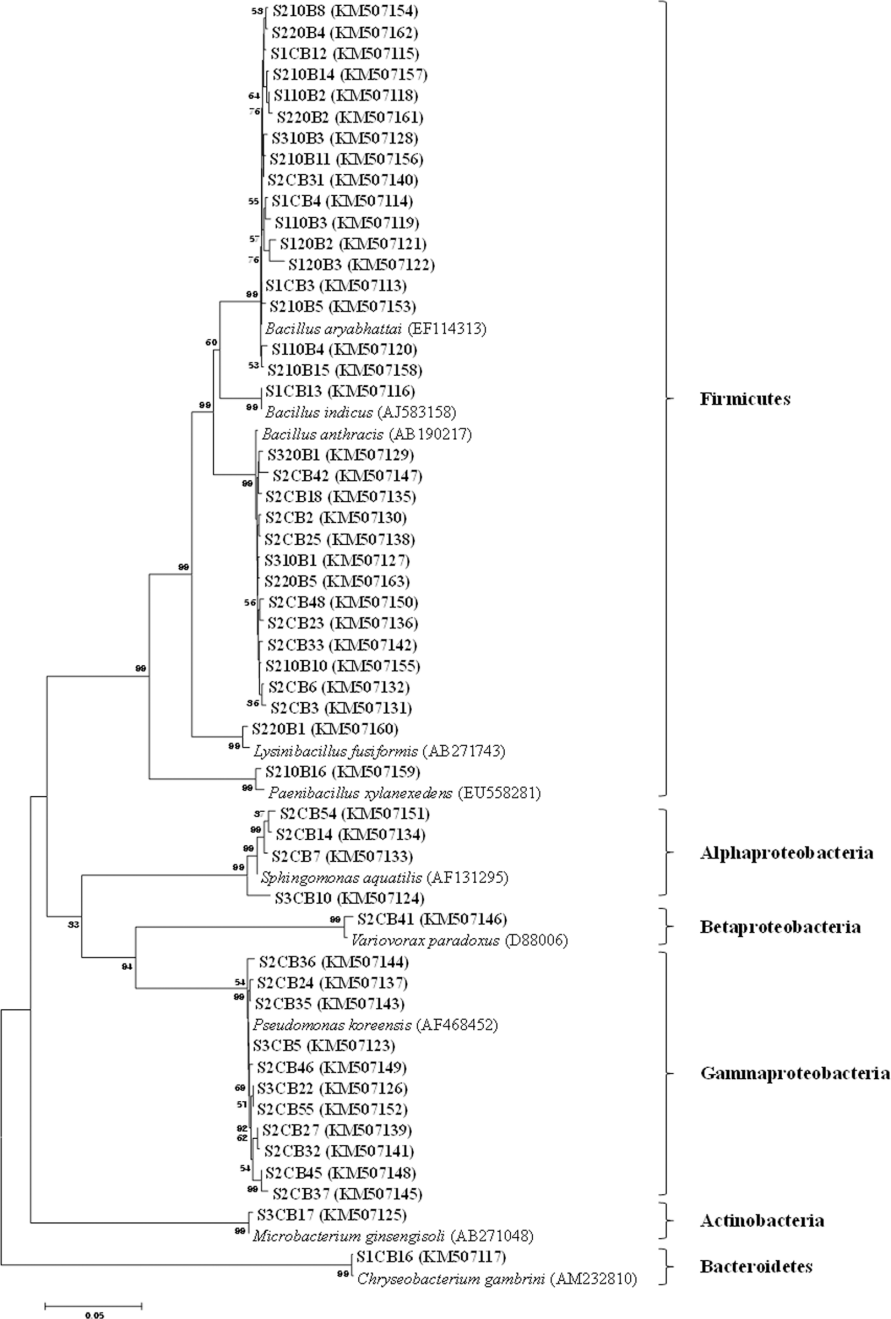
Neighbor-joining phylogenetic tree based on 16S rDNA sequence analysis, illustrating phylogenetic relationship of spore associated bacteria isolated from spore walls of AMF. SAB isolated from three types of spores at different disinfection time intervals possessing a minimum of three association characters were sequenced. The numbers at the nodes indicate the bootstrap support levels based on a neighbor joining analysis of 1000 resampled data sets. The SAB 16S rDNA sequences obtained from AMF spore are shown in bold. The numbers in the parenthesis are the nucleotide sequence accession numbers in the GenBank.

Fifty-one SAB which showed at least three spore association characters were sequenced and the closest neighbors were identified (Fig 5). The 16S rDNA sequencing revealed that the SAB associated with AMF spores belong to phyla Firmicutes, Proteobacteria, Actinobacteria and Bacteroides and genera *Bacillus*, *Lysinibacillus*, *Paenibacillus*, *Sphingomonas*, *Variovorax*, *Pseudomonas*, *Microbacterium* and *Chryseobacterium*. Nine different species from 0 min, three different species from 10 and three different species from 20 min disinfected spores were isolated (Table 1).

**Table 1:** AMF spore and its associated bacteria isolated at different time intervals of surface sterilization. The molecular identification of these SAB were chosen based on their ability to possess at least three spore association characters.

AMF spore	Spore associated bacteria isolated after		
	0 min	10 min	20 min
*Funneliformis caledonium*	• *Bacillus aryabhattai* (3) • *Bacillus indicus* (1) • *Chryseobacterium gambrini* (1)	• *Bacillus aryabhattai* (3)	• *Bacillus aryabhattai* (2)
*Racocetra alborosea*	• *Bacillus anthracis* (9) • *Pseudomonas koreensis* (9) • *Sphingomonas aquatilis* (3) • *Bacillus aryabhattai* (1) • *Variovorax paradoxus* (1)	• *Bacillus aryabhattai* (5) • *Bacillus anthracis* (1) • *Paenibacillus xylanexedens* (1)	• *Bacillus aryabhattai* (2) • *Bacillus anthracis* (1) • *Lysinibacillus fusiformis* (1)
*Funneliformis mosseae*	• *Pseudomonas koreensis* (2) • *Sphingomonas aquatilis* (1) • *Microbacterium ginsengisoli* (1)	• *Bacillus anthracis* (1) • *Bacillus aryabhattai* (1)	• *Bacillus anthracis* (1)

Number in the parenthesis indicates the number of isolates.

All the 51 SAB were able to show IAA production and the highest IAA production in the presence of tryptophan was observed in *Bacillus aryabhattai* S210B8 followed by *Microbacterium ginsengisoli* S3CB17 (S4 Table). Fifteen SAB were able to grow in 10% of NaCl, nineteen bacteria were able to grow in 6% NaCl, ten SAB were able to grow in 4% of NaCl and only seven bacteria were not able to grow in 2% NaCl. Among the 51 SAB, 24 SAB showed ACC deaminase activity, in particular, *Variovorax paradoxus* S2CB41 showed the highest activity among all the strains. Twenty one SAB were able to solubilize tricalcium phosphate. The highest P solubilizing ability was observed in *Pseudomonas koreensis* S2CB37 followed by *Paenibacillus xylanexedens* S210B16. Among 21 SAB tested, 20 SAB were able to fix atmospheric nitrogen. Only four SAB were able to produce siderophore. All of which are *Pseudomonas* species.

## Discussion

Interaction between AMF and bacteria is of considerable interest due to their beneficial effect on plant growth and yield [34,35]. AMF spore germination, viability, root colonization and spore density have been influenced by spore associated bacteria [36]. Spore associated bacteria enhance spore germination by removing the spore outer hyaline layer and inhibiting the toxic compounds that affects spore germination [11]. In the present study, SAB associated with the spore walls of *F*. *caledonium*, *R*. *alborosea* and *F*. *mosseae* were observed to vary in their bacterial attachment depending on spore morphology. The number of bacteria associated with AMF spores also varied from species to species. *R*. *alborosea* had the highest number of associated bacteria as it can reach above 400 μm in size, whereas the remaining spores reach about 300 μm in size. The highest number of SAB were obtained from 0 min disinfected spores. When disinfection time increased, the number of associated bacteria reduced showing that only strictly associated bacteria remained after 20 min of disinfection.

SEM observation of spores before and after treating them with disinfection solution revealed that there were no damage on the spore wall even after 30 min of disinfection. Our observation on the spore wall revealed the occurrence of various shapes of bacterial cells at 0, 10 min and 20 min disinfected spores. The complete surface sterilization of AMF spores after 30 min disinfection shows that the bacterial attachment on the spore wall was limited mostly to the spore outer hyaline layer [37]. Cocci-shaped bacterial cells were clearly visible on the spore walls of *F*. *caledonium* after 20 min disinfection. Ames et al. [38] observed the presence of long filament and cocci shaped bacterial colonization on the spore outer hyaline layer. We also observed that the outer hyaline layer was replaced by mucilaginous products and the reason is that the spore outer hyaline layer was sloughing off or decaying in mature spores [11].

We have used BOX-PCR based DNA fingerprints to understand the genetic similarity between the cultured SAB as it is reproducible, rapid, easy and highly discriminatory at the strain level [39]. Amplification of BOX-PCR elements provide a strain level fingerprints to find out phylogenetic relatedness among the different isolates [40]. In the present study, we found that diverse bacterial communities were associated with AMF spores and the bacterial communities belonging to the same cluster were able to associate with different AMF species. For instance, members of Bacillales order that were grouped in the same cluster were associated with all three AMF species. The same trend of association was observed by Agnolucci et al. [7] who reported that *Bacillus* and *Pseudomonas* genera were associated with *Funneliformis coronatum*, *Funneliformis mosseae*, and *Rhizophagus intraradices* regardless of AMF species.

In our study, we found that both Gram positive and Gram negative bacteria adhered with the spore wall, however, only Gram positive bacteria were able to attach or adhere more strictly with the spore walls. Other studies also reported that Gram positive bacteria were more active in bulk soil containing mycorrhiza with low-nutrient content [41] and closely associate with the external mycelium of AMF [5]. These results and our findings confirmed the proposal by Artursson et al. [34] that Gram positive bacteria are more strictly associated with AMF spores than Gram negative bacteria.

Only limited number of studies have attempted to investigate the mechanism of AMF and bacteria interaction and their associative characters. The occurrence of bacteria associated with AMF were reported to be present on AMF spore wall [42,43], hyphae [16] and AMF cytoplasm [12]. According to previous studies, it is difficult to conclude which bacteria can attach on the AMF spore walls and how closely they are associated. Lecomte et al. [16] reported that bacteria can survive by utilizing the hyphal exudates as sole nutrient source when no other nutrients are available. So far, the identified hyphal exudates such as formate, acetate, glucose and oligosaccharides can be utilized by bacteria as carbon sources [44,45], and other unidentified trace substances might also influence bacterial growth [46]. Bacteria having hydrolytic enzyme activities such as chitinase and cellulase were associated with AMF spores [14].

In our study, SAB isolated from three different AMF species were characterized for spore association characters. Hydrolytic enzyme activity may help bacteria to associate with AMF spore wall and hyphae [11,47]. Our results revealed that bacteria closely associated with *F*. *caledonium*, *R*. *alborosea* and *F*. *mosseae* were able to utilize chitin as a carbon source. In this study, we found that 74% of the bacteria associated with the spore walls of AMF had chitinolytic activity. Chitinolytic activity of bacteria also enhances AMF and plant root recognition [48] by stimulating the spores to produce more signaling molecules. In a recent study, Genre et al. [49] reported that short-chain chitin oligomers produced by *Gigaspora margarita* triggered AM-related signaling pathway by inducing the nuclear Ca^2+^ spiking in *Medicago truncatula* roots.

Chitinolytic activity of the spore associated bacteria have previously been reported. However, other possible nutrient sources present in the spore walls are not clearly understood. In our results, we found that higher percentage of SAB isolated from disinfected spores had protease activity suggesting that protease activity might play a vital role in AMF and bacteria association by enabling the bacteria to utilize the protein as a nutrient source. Likewise, high percentage of cellulolytic SAB have been found on the spore walls which is in line with Albertsen et al. [50] who reported that cellulase activity plays a significant role in AMF and bacteria association. EPS production has been reported to be involved in bacterial association with AMF spore and hyphae [34,35,51,52]. Bianciotto et al. [51] reported that mutant bacteria impaired in EPS production could not effectively associate with AMF colonized roots, whereas wild type bacteria were closely adhered. In our study, we found that SAB produce exopolysaccharide, a binding material which help the bacteria aggregate and attach to the spore walls. The number of SAB producing EPS were reduced after spore disinfection, which suggests that EPS production alone may not be sufficient for bacteria to closely associate with AMF spores under stress conditions.

Molecular identification of SAB revealed that bacteria belonging to different genera were associated with each AMF spores. Bacteria isolated from 20 min disinfected spores, *Bacillus* spp., *Paenibacillus* sp. and *Lysinibacillus* sp., were closely associated with AMF spore walls. In our study, we found that bacterial genera *Bacillus* and *Lysinibacillus* were associated with all three types of spores. The occurrence of Bacillales, Sphingomonadales, Burkholderiales, Pseudomonadales, Actinomycetales and Flavobacteriales is consistent with previous studies [7,16,53–55] suggesting that order Actinomycetales and Burkholderiales were particularly abundant in the AMF spores. *Bacillus* species are often reported to have close association with AMF spores. Several studies have reported that *Bacillus* species were associated with AMF mycelium [5], decontaminated spores walls [6] and within mycorrhizal roots [55]. The members of order Bacillales have been reported to potentially improve spore germination, hyphal growth and mycorrhizal colonization [22,56]. *Pseudomonas* species, a widespread soil bacteria, were often found to associate with AMF spores. Xavier and Germida [6] reported that *Pseudomonas* species such as *P*. *corrugata* and *P*. *fluorescens* were associated on the spore walls of *Rhizophagus clarus* (formerly—*Glomus clarum)*. *Pseudomonas* sp. isolated from the mycorrhizosphere of *Funneliformis mosseae* (formerly—*Glomus mosseae*) was able to stimulate the mycorrhizal colonization and hyphal growth of *F*. *mosseae in vitro* [57]. The most common spore associated bacteria *Variovorax paradoxus* [5,6] has been retrieved in our study as well. However, we also retrieved bacterial cultures of *Bacillus anthracis* from *Racocetra alborosea* and *Funneliformis mosseae* spores, and yet their roles in association with AMF spores are not clear. Multiple PGP characters were found in all SAB. These PGP characters of SAB may positively influence the fungal and host recognition and improve plant growth. Recently, Battini et al. [58] isolated and checked the functional diversity of cultivable bacteria associated with plant beneficial symbiont *Rhizophagus intraradices* and found that the SAB had multiple PGP characters.

## Conclusions

Fungi and bacteria are present everywhere and have been found to live side by side and interact. The range of mechanisms for both symbiosis and antagonism takes place during AMF and bacterial interaction. Our results demonstrate that bacteria which produce hydrolytic enzymes and exopolysaccharides are closely associated with AMF spore walls. Diverse bacterial communities were associated with each AMF spores. Bacteria belonging to the same species were associated with different AMF species. Bacteria belonging to orders Bacillales, Sphingomonadales, Burkholderiales, Pseudomonadales and Actinomycetales are found to be closely associated with AMF. The results of our study provide insights into the bacterial communities associated with spores of *F*. *caledonium*, *R*. *alborosea* and *F*. *mosseae*. Further molecular studies may reveal the exact mechanism for AMF and bacteria association which will bring us new insights in the field of fungal-bacterial interaction.

## Supporting Information

S1 Fig Spore morphological differentiation based on size, color and number of spore wall. (TIF)

S2 Fig SEM view of spores before and after disinfection processes. a, c, e–control spores of Type 1, 2 and 3 spore respectively, arrow indicates the sloughing outer hyaline layer of matured spores. b, d, f–sterilized spores lacking outer hyaline layer or eroded completely until some remnants left over by surface sterilization.
(TIF)

S3 Fig Genetic diversity of *Funneliformis caledonium* spore associated bacteria. (TIF)

S4 Fig Genetic diversity of *Rococetra alborosea* spore associated bacteria. (TIF)

S5 Fig Genetic diversity of *Funneliformis mosseae* spore associated bacteria. (TIF)

S6 Fig Gram staining of SAB. Means indicated with same letter do not differ significantly according to DMRT test at *P* < 0.05 (arcsine transformed data).
(TIF)

S1 Table EC and spore count of the soil samples collected from Saemangeum reclaimed land. (DOCX)

S2 Table 18S rDNA sequencing morphologically differentiated spore used for isolation of spore associated bacteria. (DOCX)

S3 Table Number of SAB isolated from three different spores at different time intervals. (DOCX)

S4 Table Plant growth promoting (PGP) characters of spore associated bacteria (SAB). (DOCX)
