# Supplementary figures and images for: Genetic Diversity and Association Characters of Bacteria Isolated from Arbuscular Mycorrhizal Fungal Spore Walls
Source: PLoS One. 2016 Aug 1;11(8):e0160356. doi: 10.1371/journal.pone.0160356 (PMC4968797; doi:10.1371/journal.pone.0160356)

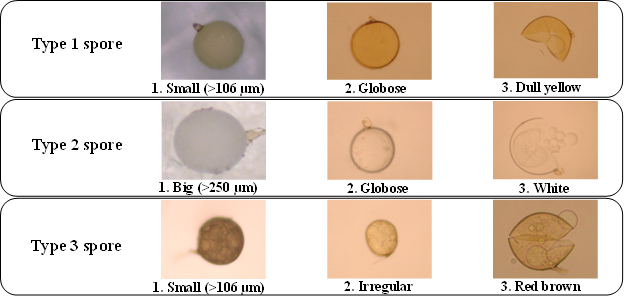

Supplement: S1 Fig — (TIF) [file pone.0160356.s001.tif]

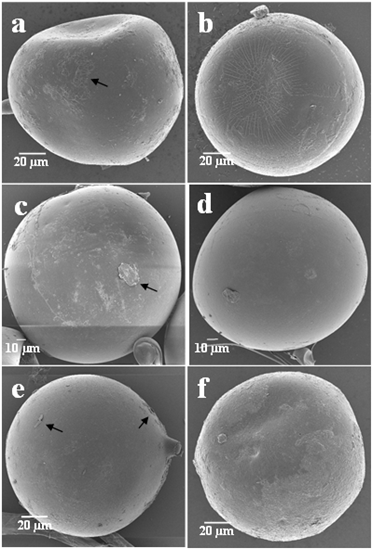

Supplement: S2 Fig — a, c, e–control spores of Type 1, 2 and 3 spore respectively, arrow indicates the sloughing outer hyaline layer of matured spores. b, d, f–sterilized spores lacking outer hyaline layer or eroded completely until some remnants left over by surface sterilization. (TIF) [file pone.0160356.s002.tif]

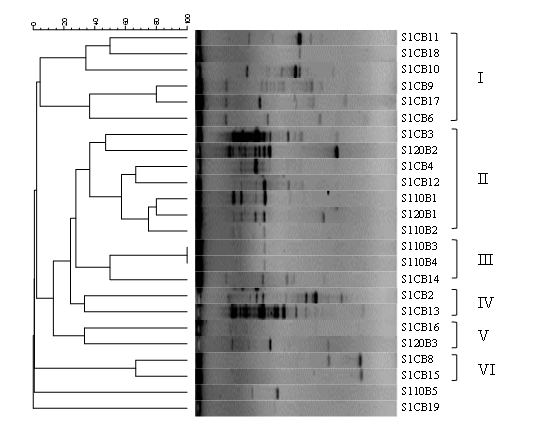

Supplement: S3 Fig — (TIF) [file pone.0160356.s003.tif]

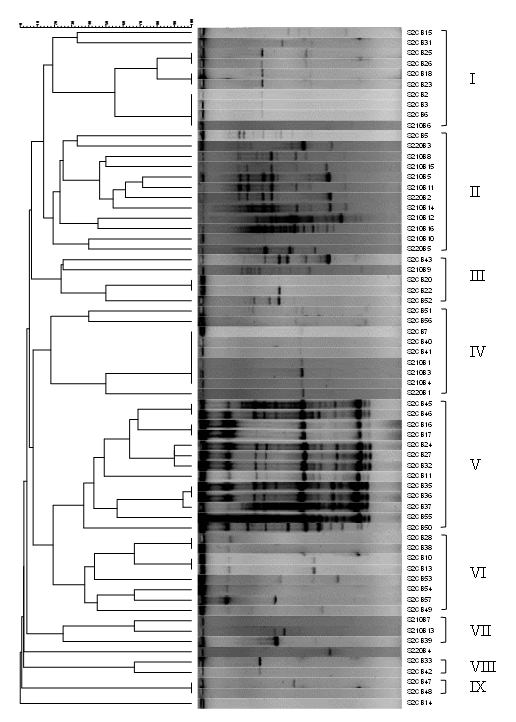

Supplement: S4 Fig — (TIF) [file pone.0160356.s004.tif]

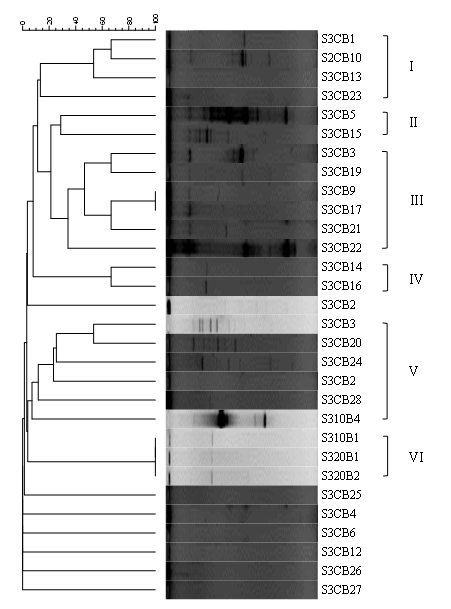

Supplement: S5 Fig — (TIF) [file pone.0160356.s005.tif]

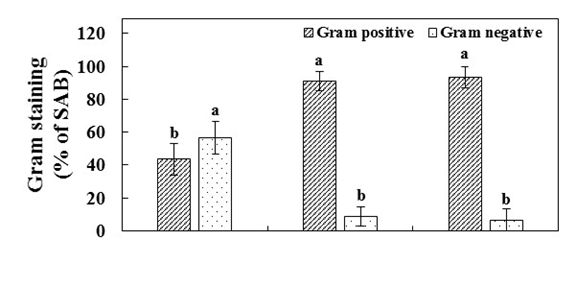

Supplement: S6 Fig — Means indicated with same letter do not differ significantly according to DMRT test at P < 0.05 (arcsine transformed data). (TIF) [file pone.0160356.s006.tif]
