# Supplementary material for: Genetic Diversity and Association Characters of Bacteria Isolated from Arbuscular Mycorrhizal Fungal Spore Walls
Source: PLoS One. 2016 Aug 1;11(8):e0160356. doi: 10.1371/journal.pone.0160356 (PMC4968797; doi:10.1371/journal.pone.0160356)
Supplement: S1 Table — (DOCX) [file pone.0160356.s007.docx]

**Table S1.** EC and spore count of the soil samples collected from Saemangeum reclaimed land

| **Sample** | **EC (dS/m)** | **pH** | **Total spores**  **(100 g of soil)** | **Healthy spores**  **(100 g of soil)** |
| --- | --- | --- | --- | --- |
| Sample 1 | 0.27 ± 0.02 | ND | 69 | 7 |
| Sample 2 | 0.30 ± 0.02 | 7.91 ± 0.1 | 233 | 22 |
| Sample 3 | 0.18 ± 0.01 | ND | 32 | 5 |
| Sample 4 | 0.32 ± 0.06 | 7.44 ± 0.04 | 73 | 11 |
| Sample 5 | 0.46 ± 0.03 | ND | 52 | 7 |
| Sample 6 | 0.13 ± 0.00 | ND | 30 | 2 |
| Sample 7 | 0.60 ± 0.03 | 6.85 ± 0.14 | 73 | 16 |
| Sample 8 | 0.18 ± 0.00 | ND | 24 | 4 |
| Sample 9 | 0.60 ± 0.04 | ND | 38 | 3 |
| Sample 10 | 0.63 ± 0.02 | ND | 54 | 4 |
| Sample 11 | 0.79 ± 0.03 | ND | 31 | 3 |
| Sample 12 | 6.92 ± 0.08 | ND | 34 | 7 |
| Sample 13 | 17.73 ± 0.02 | ND | 1 | 0 |
| Sample 14 | 8.94 ± 0.13 | ND | 16 | 2 |
| Sample 15 | 0.30 ± 0.02 | ND | 4 | 1 |
| Sample 16 | 0.43 ± 0.03 | 6.58 ± 0.03 | 107 | 63 |
| Sample 17 | 28.95 ± 2.85 | ND | 2 | 0 |
| Sample 18 | 0.79 ± 0.08 | ND | 21 | 1 |
| Sample 19 | 0.18 ± 0.01 | ND | 10 | 1 |
| Sample 20 | 0.24 ± 0.02 | 6.49 ± 0.01 | 121 | 29 |
| Sample 21 | 3.99 ± 0.52 | ND | 3 | 0 |
| Sample 22 | 14.35 ± 0.77 | ND | 5 | 1 |
| Sample 23 | 2.17 ± 0.03 | ND | 17 | 1 |
| Sample 24 | 11.81 ± 0.92 | ND | 0 | 0 |
| Sample 25 | 8.47 ± 0.36 | ND | 6 | 1 |
| Sample 26 | 36.50 ± 1.45 | ND | 0 | 0 |
| Sample 27 | 18.97 ± 0.29 | ND | 1 | 0 |
| Sample 28 | 9.29 ± 0.10 | ND | 2 | 1 |
| Sample 29 | 0.76 ± 0.04 | ND | 4 | 1 |
| Sample 30 | 10.16 ± 0.6 | ND | 11 | 2 |
| Sample 31 | 0.34 ± 0.02 | 6.54 ± 0.07 | 16 | 12 |
| Sample 32 | 0.36 ± 0.05 | 6.25 ± 0.03 | 218 | 161 |
| Sample 33 | 1.60 ± 0.22 | 6.2 ± 0.02 | 76 | 63 |
| Sample 34 | 1.40 ± 0.04 | 6.32 ± 0.02 | 64 | 36 |
| Sample 35 | 1.13 ± 0.08 | 6.36 ± 0.04 | 20 | 15 |

ND – Not determined.
