# Supplementary material for: Genetic Diversity and Association Characters of Bacteria Isolated from Arbuscular Mycorrhizal Fungal Spore Walls
Source: PLoS One. 2016 Aug 1;11(8):e0160356. doi: 10.1371/journal.pone.0160356 (PMC4968797; doi:10.1371/journal.pone.0160356)
Supplement: S2 Table — (DOCX) [file pone.0160356.s008.docx]

**Table S2.** 18S rDNA sequencing morphologically differentiated spore used for isolation of spore associated bacteria.

| Strain | Closest match | Base pair length | Max identity | Accession No. |
| --- | --- | --- | --- | --- |
| Type 1 spore | *Funneliformis caledonium* | 477 bp | 99% | KJ792101 |
| Type 2 spore | *Racocetra alborosea* | 470 bp | 100% | KJ792102 |
| Type 3 spore | *Funneliformis mosseae* | 478 bp | 100% | KJ792103 |
