# Supplementary material for: Genetic Diversity and Association Characters of Bacteria Isolated from Arbuscular Mycorrhizal Fungal Spore Walls
Source: PLoS One. 2016 Aug 1;11(8):e0160356. doi: 10.1371/journal.pone.0160356 (PMC4968797; doi:10.1371/journal.pone.0160356)
Supplement: S3 Table — (DOCX) [file pone.0160356.s009.docx]

**Table S3.** Number of SAB isolated from three different spores at different time intervals

| AMF identification | SAB isolated after | | | | Total |
| --- | --- | --- | --- | --- | --- |
|  | 0 min | 10 min | 20 min | 30 min |  |
| *Funneliformis caledonium* | 16 | 5 | 3 | 0 | 24 |
| *Racocetra alborosea* | 46 | 15 | 5 | 0 | 66 |
| *Funneliformis mosseae* | 24 | 4 | 2 | 0 | 30 |
| Total | 86 | 24 | 10 | 0 | 120 |
