# Supplementary material for: Genetic Diversity and Association Characters of Bacteria Isolated from Arbuscular Mycorrhizal Fungal Spore Walls
Source: PLoS One. 2016 Aug 1;11(8):e0160356. doi: 10.1371/journal.pone.0160356 (PMC4968797; doi:10.1371/journal.pone.0160356)
Supplement: S4 Table — (DOCX) [file pone.0160356.s010.docx]

**Table S4.** Plant growth promoting (PGP) characters of spore associated bacteria (SAB)

| Strain | Salt tolerance  (%) | IAA production^a^ | | ACC deaminase  activity^b^ | P solubilization^c^ | N fixation | Siderophore  production |
| --- | --- | --- | --- | --- | --- | --- | --- |
|  |  | Without tryptophan | With tryptophan |  |  |  |  |
| *Bacillus aryabhattai* S1CB3 | >10 | 1.31 ± 0.01 | 3.46 ± 0.66 | 3.12 ± 0.28 | 15.03 ± 4.94 | + | - |
| *Bacillus aryabhattai* S1CB4 | >10 | 1.34 ± 0.01 | 5.64 ± 1.74 | 14.1 ± 7.09 | 2.13 ± 0.04 | + | - |
| *Bacillus aryabhattai* S1CB12 | >10 | 1.32 ± 0.00 | 5.79 ± 1.67 | 00.0 ± 0.00 | ND | ND | ND |
| *Bacillus indicus* S1CB13 | 6 | 1.32 ± 0.01 | 1.71 ± 0.04 | 00.0 ± 0.00 | ND | ND | ND |
| *Chryseobacterium gambrini* S1CB16 | 2 | 1.34 ± 0.00 | 3.54 ± 0.24 | 00.0 ± 0.00 | ND | ND | ND |
| *Bacillus aryabhattai* S110B2 | >10 | 1.34 ± 0.01 | 3.24 ± 0.58 | 10.52 ± 2.43 | 29.68 ± 8.61 | + | - |
| *Bacillus aryabhattai* S110B3 | 6 | 1.31 ± 0.07 | 6.32 ± 1.52 | 2.37 ± 1.48 | 22.92 ± 6.15 | + | - |
| *Bacillus aryabhattai* S110B4 | 6 | 1.36 ± 0.02 | 3.77 ± 0.41 | 1.35 ± 0.65 | 0.53 ± 0.04 | + | - |
| *Bacillus aryabhattai* S120B2 | >10 | 2.12 ± 0.08 | 2.52 ± 0.24 | 2.68 ± 1.57 | 50.9 ± 16.28 | + | - |
| *Bacillus aryabhattai* S120B3 | >10 | 1.29 ± 0.00 | 2.11 ± 0.19 | 4.14 ± 3.49 | 22.04 ± 4.03 | + | - |
| *Bacillus anthracis* S2CB2 | 6 | 1.96 ± 0.12 | 3.13 ± 0.13 | 00.0 ± 0.00 | ND | ND | ND |
| *Bacillus anthracis* S2CB3 | 6 | 1.99 ± 0.03 | 2.87 ± 0.12 | 00.0 ± 0.00 | ND | ND | ND |
| *Bacillus anthracis* S2CB6 | 6 | 2.34 ± 0.22 | 2.97 ± 0.12 | 00.0 ± 0.00 | ND | ND | ND |
| *Sphingomonas aquatilis* S2CB7 | 2 | 1.58 ± 0.02 | 2.44 ± 0.27 | 00.0 ± 0.00 | ND | ND | ND |
| *Sphingomonas aquatilis* S2CB14 | 2 | 1.78 ± 0.02 | 2.57 ± 0.16 | 00.0 ± 0.00 | ND | ND | ND |
| *Bacillus anthracis* S2CB18 | 6 | 1.52 ± 0.03 | 3.38 ± 0.35 | 00.0 ± 0.00 | ND | ND | ND |
| *Bacillus anthracis* S2CB23 | 4 | 1.96 ± 0.34 | 4.44 ± 0.42 | 00.0 ± 0.00 | ND | ND | ND |

| Strain | Salt tolerance  (%) | IAA production^a^ | | ACC deaminase  activity^b^ | P solubilization^c^ | N fixation | Siderophore  production |
| --- | --- | --- | --- | --- | --- | --- | --- |
|  |  | Without tryptophan | With tryptophan |  |  |  |  |
| *Pseudomonas koreensis* S2CB24 | 4 | 2.06 ± 0.08 | 7.38 ± 0.27 | 00.0 ± 0.00 | ND | ND | ND |
| *Bacillus anthracis* S2CB25 | 6 | 1.98 ± 0.06 | 4.46 ± 0.19 | 00.0 ± 0.00 | ND | ND | ND |
| *Pseudomonas koreensis* S2CB27 | 4 | 1.98 ± 0.1 | 6.77 ± 0.18 | 00.0 ± 0.00 | ND | ND | ND |
| *Bacillus aryabhattai* S2CB31 | >10 | 1.33 ± 0.03 | 2.38 ± 0.41 | 4.06 ± 1.17 | 76.43 ± 27.73 | + | - |
| *Pseudomonas koreensis* S2CB32 | 4 | 2.00 ± 0.04 | 6.43 ± 0.04 | 0.65 ± 0.34 | 105.64 ± 2.33 | + | + |
| *Bacillus anthracis* S2CB33 | 4 | 2.11 ± 0.20 | 3.87 ± 0.72 | 00.0 ± 0.00 | ND | ND | ND |
| *Pseudomonas koreensis* S2CB35 | 4 | 2.01 ± 0.03 | 8.47 ± 0.73 | 0.78 ± 0.48 | 108.54 ± 2.88 | + | + |
| *Pseudomonas koreensis* S2CB36 | 4 | 2.31 ± 0.16 | 7.11 ± 0.50 | 00.0 ± 0.00 | ND | ND | ND |
| *Pseudomonas koreensis* S2CB37 | 4 | 2.05 ± 0.07 | 6.34 ± 0.14 | 2.38 ± 1.39 | 113.1 ± 1.31 | + | + |
| *Variovorax paradoxus* S2CB41 | 2 | 1.93 ± 0.11 | 1.91 ± 0.07 | 14.5 ± 0.16 | ND | ND | ND |
| *Bacillus anthracis* S2CB42 | 6 | 1.69 ± 0.04 | 1.33 ± 0.02 | 11.54 ± 1.03 | 104.68 ± 0.46 | + | - |
| *Pseudomonas koreensis* S2CB45 | 6 | 2.01 ± 0.04 | 6.16 ± 0.14 | 1.65 ± 0.53 | 108.18 ± 1.52 | + | + |
| *Pseudomonas koreensis* S2CB46 | 6 | 1.62 ± 0.02 | 2.85 ± 0.05 | 00.0 ± 0.00 | ND | ND | ND |
| *Bacillus anthracis* S2CB48 | 6 | 2.24 ± 0.12 | 4.91 ± 0.37 | 00.0 ± 0.00 | ND | ND | ND |
| *Sphingomonas aquatilis* S2CB54 | 2 | 0.83 ± 0.42 | 2.87 ± 0.06 | 2.13 ± 0.47 | ND | ND | ND |
| *Pseudomonas koreensis* S2CB55 | 4 | 1.90 ± 0.03 | 6.57 ± 0.09 | 00.0 ± 0.00 | ND | ND | ND |
| *Bacillus aryabhattai* S210B5 | >10 | 2.26 ± 0.07 | 5.47 ± 0.11 | 00.0 ± 0.00 | ND | ND | ND |

| Strain | Salt tolerance  (%) | IAA production^a^ | | ACC deaminase  activity^b^ | P solubilization^c^ | N fixation | Siderophore  production |
| --- | --- | --- | --- | --- | --- | --- | --- |
|  |  | Without tryptophan | With tryptophan |  |  |  |  |
| *Bacillus aryabhattai* S210B8 | >10 | 1.63 ± 0.03 | 10.95 ± 2.08 | 2.46 ± 2.46 | 79.85 ± 2.45 | + | - |
| *Bacillus anthracis* S210B10 | 6 | 2.08 ± 0.00 | 4.64 ± 0.07 | 00.0 ± 0.00 | ND | ND | ND |
| *Bacillus aryabhattai* S210B11 | >10 | 1.88 ± 0.19 | 6.37 ± 1.30 | 1.95 ± 1.95 | 108.27 ± 1.47 | + | - |
| *Bacillus aryabhattai* S210B14 | >10 | 1.41 ± 0.04 | 2.31 ± 0.14 | 00.0 ± 0.00 | ND | ND | ND |
| *Bacillus aryabhattai* S210B15 | >10 | 1.85 ± 0.05 | 5.21 ± 0.52 | 2.59 ± 2.14 | 34.94 ± 5.96 | + | - |
| *Paenibacillus xylanexedens* S210B16 | 6 | 0.78 ± 0.39 | 1.67 ± 0.04 | 4.48 ± 4.48 | 109.32 ± 1.21 | - | - |
| *Lysinibacillus fusiformis* S220B1 | 6 | 1.99 ± 0.13 | 7.08 ± 0.34 | 00.0 ± 0.00 | ND | ND | ND |
| *Bacillus aryabhattai* S220B2 | >10 | 1.27 ± 0.04 | 3.21 ± 0.68 | 1.12 ± 1.12 | 94.32 ± 1.21 | + | - |
| *Bacillus aryabhattai* S220B4 | >10 | 1.54 ± 0.05 | 3.17 ± 0.69 | 2.11 ± 1.39 | 95.90 ± 0.70 | + | - |
| *Bacillus anthracis* S220B5 | 6 | 2.01 ± 0.03 | 4.28 ± 0.47 | 1.69 ± 1.00 | 69.41 ± 2.02 | + | - |
| *Pseudomonas koreensis* S3CB5 | 4 | 1.35 ± 0.00 | 6.07 ± 0.23 | 00.0 ± 0.00 | ND | ND | ND |
| *Sphingomonas aquatilis* S3CB10 | 2 | 2.81 ± 0.09 | 2.63 ± 0.21 | 8.15 ± 1.56 | ND | ND | ND |
| *Microbacterium ginsengisoli* S3CB17 | 2 | 1.63 ± 0.01 | 10.46 ± 0.08 | 00.0 ± 0.00 | ND | ND | ND |
| *Pseudomonas koreensis* S3CB22 | 6 | 1.99 ± 0.01 | 6.09 ± 0.13 | 00.0 ± 0.00 | ND | ND | ND |
| *Bacillus anthracis* S310B1 | 6 | 2.35 ± 0.12 | 3.82 ± 1.06 | 00.0 ± 0.00 | ND | ND | ND |
| *Bacillus aryabhattai* S310B3 | >10 | 2.71 ± 0.30 | 6.98 ± 3.63 | 1.8 ± 1.22 | 26.25 ± 1.75 | + | - |
| *Bacillus anthracis* S320B1 | 6 | 2.41 ± 0.14 | 3.91 ± 0.27 | 00.0 ± 0.00 | ND | ND | ND |

Each value represents the mean of 3 replications ± SE. ^a^µg ml^-1^; ^b^µmol α-Ketobutyrate (mg protein)^-1^ h^-1^; ^c^g l^-1^; ‘+’ – positive; ‘-‘ – negative; ND – Not Determined.
